# Supplementary material for: An open-source framework for large-scale, flexible evaluation of biomedical text mining systems
Source: J Biomed Discov Collab. 2008 Jan 29;3:1. doi: 10.1186/1747-5333-3-1 (PMC2276192; doi:10.1186/1747-5333-3-1)
Supplement: Additional file 1 — Evaluation platform source code. The additional file contains the software evaluation framework discussed in this paper. The most current release can be downloaded from SourceForge.net [1]. See the accompanying README file for instructions on installation and use. Also included in the distribution is a collection of UIMA wrappers for some commonly used BioNLP tools and annotated corpora. [file 1747-5333-3-1-S1.zip › bionlp_uima_repository-1.0.1/docs/api/src-html/edu/uchsc/ccp/util/nlp/mention/comparison/MentionComparator.html]

```
001    package edu.uchsc.ccp.util.nlp.mention.comparison;
002    
003    import java.util.Comparator;
004    import java.util.List;
005    
006    import edu.uchsc.ccp.util.nlp.annotation.comparison.SpanComparator;
007    import edu.uchsc.ccp.util.nlp.annotation.comparison.StrictSpanComparator;
008    import edu.uchsc.ccp.util.nlp.mention.ComplexSlotMention;
009    import edu.uchsc.ccp.util.nlp.mention.Mention;
010    import edu.uchsc.ccp.util.nlp.mention.SlotMention;
011    
012    /**
013     * This is an abstract class for mention comparators. Each level of comparison stops at the class mention level.<br>
014     * Level 0: comparison of the classmention names.<br>
015     * Level 1: comparison of the classmention names, and optionally their text annotation spans, and if they match, a comparison of the slotmentions and
016     * their slot values, and a comparison of complexslotmentions and their classmentionnames<br>
017     * Level 2: Comparison of the class mentions in level in the previous level that were only compared by mention name<br>
018     * ...<br>
019     * Level N: Comparison of the class mentions in level in the previous level that were only compared by mention name<br>
020     * <br>
021     * Example:<br>
022     * TA1<br>
023     * --- CM1<br>
024     * ------ SM1a<br>
025     * ---------SV1a<br>
026     * ------ SM1b<br>
027     * ---------SV1b<br>
028     * ------ CSM1a<br>
029     * --------- CM2 ==> TA2<br>
030     * ------------ SM2a<br>
031     * --------------- SV2a<br>
032     * ------------ CSM2a<br>
033     * --------------- CM3 ==> TA3<br>
034     * ------ CSM1b<br>
035     * --------- CM4 ==> TA4<br>
036     * ------------ CSM4a<br>
037     * -------------- CM5 ==> TA5<br>
038     * <br>
039     * Level 0: TA1 span and CM1 mention name compared<br>
040     * Level 1: SM1a name and values, SM1b name and values, CM2 name, TA2 span, CM4 name, and TA4 span are also compared<br>
041     * Level 2: SM2a name and values, CM3 name, TA3 span, CM5 name, TA5 span<br>
042     * Level 3: CM3 and CM5 slots if there are any <br>
043     * 
044     * 
045     * 
046     * @author williamb
047     * 
048     */
049    public abstract class MentionComparator implements Comparator<Mention> {
050    
051        /**
052         * So that differences found in mentions can be distinguished between differences found in spans, the mention comparators should return -3, 0, or
053         * 3, therefore the MULTIPLIER variable is used to scale the compare() output.
054         */
055        protected final int MULTIPLIER = 3;
056    
057        /**
058         * The default use of compare() will compare all depth levels of the mention heirarchy, and will compare any linked TextAnnotations using the
059         * StrictSpanComparator. This is specified by a maximum depth of -1.<br>
060         * <br>
061         * So that differences found in mentions can be distinguished between differences found in spans, the mention comparators should return -3, 0, or
062         * 3 (instead of -1, 0, or 1)
063         * 
064         * @param mention1
065         * @param mention2
066         */
067        public int compare(Mention mention1, Mention mention2) {
068            return compare(mention1, mention2, new StrictSpanComparator(), -1, 0);
069        }
070    
071        public abstract int compare(Mention mention1, Mention mention2, SpanComparator spanComparator, int maximumComparisonDepth, int depth);
072    
073        public abstract boolean hasEquivalentMentionNames(Mention mention1, Mention mention2);
074    
075        /**
076         * This method compares two lists of slot mentions by iterating through them. A quick check of the number of slot mentions in each list is made at
077         * first to see if the individual comparisons can be avoided.
078         */
079        protected boolean equalSlotMentions(List<SlotMention> smList1, List<SlotMention> smList2, SpanComparator spanComparator,
080                SlotMentionComparator slotMentionComparator, int maximumComparisonDepth, int depth) {
081            if (smList1.size() != smList2.size()) {
082                /* A quick check of the count of slot mentions */
083                return false;
084            } else {
085                /*
086                 * iterate through each slot mention and see if it has a match in the other list. We must iterate because it is possible that there could
087                 * be two slot mentions with identical names. This is not ideal, but it is possible
088                 */
089                for (SlotMention sm : smList1) {
090                    boolean slotMentionHasAMatch = false;
091                    for (SlotMention smToCompare : smList2) {
092                        if (slotMentionComparator.compare(sm, smToCompare, spanComparator,maximumComparisonDepth, depth) == 0) {
093                            slotMentionHasAMatch = true;
094                        }
095                    }
096                    if (!slotMentionHasAMatch) {
097                        return false;
098                    }
099                }
100                /* if we have made it this far, then all slot mentions have a match */
101                return true;
102            }
103        }
104    
105        /**
106         * This method compares two lists of complex slot mentions by iterating through them.
107         */
108        protected boolean equalComplexSlotMentions(List<ComplexSlotMention> csmList1, List<ComplexSlotMention> csmList2, SpanComparator spanComparator,
109                ComplexSlotMentionComparator csmComparator, int maximumComparisonDepth, int depth) {
110            if (csmList1.size() != csmList2.size()) {
111                /* a quick check of the count of the complex slot mentions */
112                return false;
113            } else {
114                /* iterate through each CSM and see if it has a match */
115                for (ComplexSlotMention csm : csmList1) {
116                    boolean csmHashAMatch = false;
117                    for (ComplexSlotMention csmToCompare : csmList2) {
118                        if (csmComparator.compare(csm, csmToCompare, spanComparator, maximumComparisonDepth, depth) == 0) {
119                            csmHashAMatch = true;
120                        }
121                    }
122                    if (!csmHashAMatch) {
123                        return false;
124                    }
125                }
126                /* if we have made it this far, then all complex slot mentions have a match */
127                return true;
128            }
129    
130        }
131    }
```
